# Design of Experiments as a Tool to Optimize the Process of Coating Minitablets with Commercial Gastro-Resistant Coating Mixtures

**DOI:** 10.3390/pharmaceutics14091816

**Published:** 2022-08-29

**Authors:** Maja Frankiewicz, Małgorzata Sznitowska

**Affiliations:** Department of Pharmaceutical Technology, Medical University of Gdansk, Hallera 107, 80-416 Gdansk, Poland

**Keywords:** design of experiments, quality by design, fluid bed coating, minitablets, enteric coating

## Abstract

According to the Quality by Design (QbD) concept, Design of Experiment (DoE) was used to indicate critical process parameters and optimize the fluid bed coating of minitablets in a laboratory size batch. Full factorial design was employed to increase knowledge of the process for three kinds of minitablet (MT) cores using two commercial gastro-resistant coating mixtures. The statistical analysis showed that different critical process parameters were indicated for the tested minitablets: X3: the coating mixture flow rate for MTs with pantoprazole sodium and Eudragit L; X2: the product temperature; X3 and X4: the spraying pressure for MTs with pantoprazole sodium and Acryl Eze II; and X1 and X2: MTs with diclofenac sodium. Such differences were the result of features, such as the sub-coat, size, and mass of the cores and the core and coating mixture composition. No optimal parameters were found for any of the tested MT types. Therefore, DoE should be considered as a statistical tool to individually optimize the process for the product, equipment, and tested parameters. However, optimization of the fluid bed coating allowed us to predict the values of the process parameters necessary to obtain good-quality products. Therefore, fluid bed coating may be successfully used to obtain modified-release MTs of high quality after applying the statistical tool DoE.

## 1. Introduction

Quality is a parameter of superior value for all regulatory bodies dealing with pharmaceutical products. Early detection of problems resulting from poorly controlled conditions may facilitate cost- and quality-effective processes. Therefore, to avoid failure, the quality of products and services must be built through proper planning. According to this principle, a method of Quality by Design (QbD) was created and described in guidelines of the International Conference on Harmonization (ICH Q8 R2) [1]. Today, implementation of this concept has become a necessity. Moreover, specification and manufacturing process control has become the standard in recent years. Based on knowledge from numerous pharmaceutical studies and from manufacturing experience, a quality risk management method was successfully developed [2].

Mathematically describing the relationships between tested factors and the quality of a product involves using a statistical tool known as “experimental design”. Assurance of quality can be achieved by combining material attributes and process parameters. These experimental plans, in an organized way, allow one to define, e.g., critical process parameters that determine product quality. Moreover, plans known as Design of Experiments (DoE) allow one to optimize these parameters and to achieve precise identification of existing interactions [3]. DoE allows the use of different mathematical models, such as Box Behnken, full and fractional factorial designs, Placke–Burman, surface design, and Taguchi [2]. Pharmaceutical companies willingly use full and fractional factorial designs based on available DoE models. Fractional designs are limited to only some experiments and are thus fast to perform but enable one to study only the main effects of the examined parameters to determine the response values representing the quality of a product. However, full factorial designs include a series of experiments to assess combinations of all tested parameters. Thus, this method is time and cost intensive but provides the most data about the process without the risk of missing important interactions. These methods are especially useful in the early stages of experimental work [4].

The selected active substances have clinical applications in the treatment of children, but the lack of an appropriate form makes it impossible to administer the drug to the youngest patients. The safety of pantoprazole for the short-term treatment of erosive esophagitis and reflux was proven in patients older than one year of age, but there is no appropriate pediatric formulation for patients less than five years old. Diclofenac is a non-steroidal anti-inflammatory drug generally used in children at least six years old who can swallow a tablet. However, in juvenile idiopathic arthritis, this drug is licensed for children above one year old. The oral dose of diclofenac sodium for juvenile idiopathic arthritis is 1 to 3 mg/kg daily in divided doses, up to a maximum of 150 mg daily. Therefore, to start, a single dose of5 mg of diclofenac can be taken two times per day and then increased with the child’s age up to 75 mg. From a caregiver’s point of view, it is impossible to measure the smallest doses based on the drugs available on the market.

Minitablets (MTs) are defined as tablets with diameters from 1 to 3 mm [5]. The small size of the tablets makes them easy to swallow and widely accepted by children of all ages [6,7]. MTs have become an innovative oral pediatric drug, and administrating MTs with variable numbers of subunits facilitates the selection of a dose appropriate to the age of the patient [8]. Furthermore, MT coatings can be used to improve flavor or provide modified-release forms, which are still a challenge in pediatric drugs [9].

The fluid bed system is a technique applied on an everyday basis in industry, mainly for granulation and coating. The coating proceeds by successive wetting of the fluidized solids with sprayed liquid followed by film forming through drying. Both the efficiency of the fluid bed coating process and the quality of the film depend on mechanism comprehension. However, the diversity of parameters used in the process, which may affect the product quality, cause difficulties in understanding these mechanisms [10].

Despite the fact that numerous publications have already reported on fluid bed coatings [9,11,12], to date, knowledge on the critical process parameters and methods for the quality control of coated MTs is negligible. Thus, there is a demand for research focused on defining the relationships between process circumstances and coated products. Thus, DoE may be helpful to deeply understand the coating process via mathematical and graphic descriptions of the observed relationships [13]. Both evaluated coating mixtures are commonly used with the chosen active substances to produce modified-release forms. Due to the fact that these are well-known mixtures without drug interactions, their use allowed us to focus on the coating process itself.

Since MTs are a novel form of pediatric drugs, research on their related technologies is an attractive topic. Furthermore, there is still no available modified-release forms that allow appropriate dosing of the drug for the youngest patients. Therefore, the main aim of this study was to develop enteric-coated MTs with the QbD concept. DoE was used to examine the impact of fluid bed coating parameters on the quality of enteric-coated MTs and optimize the process. Moreover, different cores and gastro-resistant coating mixtures were used to evaluate DoE as a universal tool to indicate critical process parameters and optimize the fluid bed coating of MTs.

## 2. Materials and Methods

### 2.1. Materials

Active pharmaceutical ingredients (APIs) were kindly donated as follows: pantoprazole sodium sesquihydrate was donated by LEK-AM (Zakroczym, Poland) and diclofenac sodium was donated by Polpharma SA (StarogardGdanski, Poland). Colloidal silicon dioxide: Aerosil 200 (from Evonik, Darmstadt, Germany); crospovidone: Kollidon CLF (from BASF, Ludwigshafen, Germany); lactose monohydrate: Flowlac 100 and GranuLac 200 (from Meggle, Wasserburg, Germany); microcrystalline cellulose: Avicel PH101 (from Sigma-Aldrich, Steinheim, Germany) and Vivapur PH102 (from JRS Pharma, Rosenberg, Germany); pregelatinized maize starch: Starch 1500 (from Colorcon, Dartford, UK); sodium carbonate (from PPH STANLAB, Lublin, Poland); sodium starch glycolate: Vivastar P (from JRS PHARMA, Rosenberg, Germany); and sodium stearyl fumarate: PRUV (from JRS PHARMA, Rosenberg, Germany) were used to compose the MT cores. The sub-coating was made from hypromellose: Pharmacoat 606 (from Shin-Etsu Chemical, Tokyo, Japan) and polyethylene glycol 6000 (from Sigma-Aldrich, Steinheim, Germany). A methacrylic acid-ethyl acrylate copolymer was used to obtain the enteric film: Eudragit L 30D55 (from Evonik Industries, Darmstadt, Germany) and the ready-to-use mixture Acryl Eze II (from Colorcon, Dertford, UK). Eudragit was mixed with triethyl citrate (from Sigma-Aldrich, Steinheim, Germany) and talc (from Luzenac VAL Chisone, Porte, Italy).

### 2.2. Methods

#### 2.2.1. Production and Physical Evaluation of Minitablet Cores

Both tablet masses were prepared via high-shear wet granulation in a granulator Prymus from Zelmer SA (Rzeszow, Poland). The composition of MTs and process conditions are presented in Table 1. Pantoprazole sodium was granulated with a 25% (*w*/*w*) solution of sodium carbonate, whereas diclofenac sodium, fillers, and a binder were granulated with water. The granules were sieved through a 1.2 (pantoprazole) and a 1.0 mm (diclofenac) mesh and dried to obtain 4% moisture content. The granule size distribution was estimated via sieve analysis. For pantoprazole granules, the main obtained fraction was 800–560 µm, and for diclofenac the main fraction was 560–355 µm. After granulation, the other excipients were added. A rotary tablet press, RTP-D8, from Erweka (Heusenstamm, Germany), equipped with 3 mm of diameter single punches, was used to compress biconvex MTs. The tablet masses were fed into dies using gravity as a driving force. A texture analyzer TA.XT Plus (Stable Micro Systems, Godalming, UK) was used to measure the crushing resistance of MTs (n = 10). Conforming to the European Pharmacopoeia (PhEur 10.0; 2.9.7), de-dusted MTs (6.5 g) were subjected to friability tests. The cores were placed for 4 min in a TAR-10 friability tester from Erweka (Heusenstamm, Germany). After 100 rotations (25 rotation per minute), the loss of mass was calculated.

#### 2.2.2. Production and Evaluation of Enteric-Coated Minitablets

An Aircoater 025 InnoJet (Romaco, Steinen, Germany) was used for the fluid bed coating of MTs. A sub-coating was applied on the MTs with pantoprazole. A solution of hypromellose with polyethylene glycol 6000 (concentration 10% *w*/*w* in 9:1 ratio) was sprayed until obtaining a 60 µm film thickness (samples of the product were tested at line using the stereoscopic method). Gastro-resistant API was achieved by spraying the MT cores with an aqueous dispersion of a pH-sensitive coating mixture based on a methacrylic acid-ethyl acrylate copolymer (1:1), which is soluble only at pH values higher than 5.5. MTs with pantoprazole were coated using Eudragit L and Acryl Eze II, and two final products were obtained: MT-PE and MT-PA, respectively. However, MTs with diclofenac were coated only with Eudragit L to yield the third product, MT-DE. As recommended by the manufacturer, plasticizer (1.25% *w*/*w* of triethyl citrate), anti-adhesive agent (6.25% *w*/*w* of talc), and water (50.8% *w*/*w*) were added to the Eudragit L dispersion [14], but only water (80.0% *w*/*w*) was added to the Acryl Eze II powder (containing poloxamer 407 and talc) [15]. Both coating mixtures were prepared in a 20% concentration. 

The parameters are presented in Table 2. The fluid bed coating was performed under a constant temperature of 25 or 27 °C (with accuracy of ±0.2 °C dueto additional sensors). Sixteen lab-scale experiments (50 g batch) were performed for each product. Fixed amounts of the coating mixtures (30 g) were used for each experiment. 

**Figure 1:**
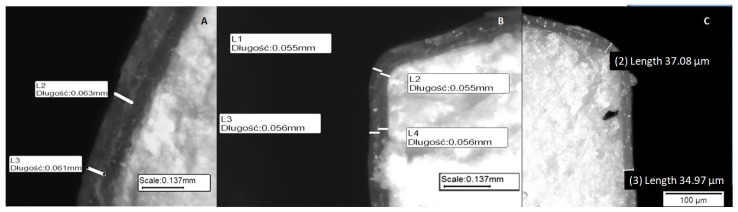
Example images of minitablet (MT) cross-sections obtained using a stereoscopic microscope: (**A**) MT-PE, (**B**) MT-PA, (**C**) MT-DE.

Coated MTs from each experiment were observed under reflected light using a stereoscopic microscope X2000 from Opta-Tech (Warszawa, Poland). The thickness of the obtained film was measured on smooth cross-sections achieved via cutting with a scalpel. Figure 1 shows example microscopic images of the MT cross-sections. The mean value of the film thickness (Z1 response) was measured in 10 positions of the cross-sections in at least five MTs of each batch.

#### 2.2.3. In Vitro Dissolution Test

According to a previous monograph, Pantoprazole Sodium Delayed-Release Tablets (PhEur 10.0), in vitro dissolution tests for MT with pantoprazole were performed using an Apparatus 1 DT 720 Series device from Erweka (Heusenstamm, Germany) with a speed of 100 rotations per minute (rpm).

For MT with diclofenac Apparatus 2, a rotating speed of 50 rpm was used according to recommendations in Diclofenac Sodium Delayed-Release Tablets (USP 43-NF 38/USP–NF 2021). All tests were performed at a constant temperature of 37 ± 0.5 °C and the volume of the dissolution medium (900 mL). The first stage of the tests (acid phase) was performed for 2 h in 0.1 N HCl. Following the first stage, the dissolution medium was changed into a 0.05 M potassium phosphate buffer (pH 6.8), and we started the second stage (buffer phase), which was carried out for 45 min. The content of API released was established inline using a UV–VIS spectrometer from Agilent (Santa Clara, CA, USA) at a 288 (pantoprazole) or 276 nm (diclofenac) wavelength. Based on the calibration curves, the mean API content (n = 6) was counted, and the method was validated. The outcomes were then compared with the PhEur 10.0 (2.9.3) requirements.

#### 2.2.4. Statistical Analysis of the Fluid Bed Coating Process

DoE was used as a statistical tool to analyze the MT coating process. The Statistica 13 software (TIBCO Software Inc., Palo Alto, CA, USA) facilitated analysis of variance for the experimental data and presented the data both mathematically and graphically. A full factorial design on two levels was applied to construct a polynomial model showing the relationship between the independent variables (X) and dependent variables (Z). Previous experience with fluid bed coatings of MTs [16] and the available literature [17] highlighted four process parameters to examine as independent variables. These variables are described in Table 2 (factors X1–X4). The low and high values of the examined parameters were established in previous experimental work [18,19]. The response values of Z1–Z3 were indicated as dependent variables to describe both the yield of the process and the quality of the coated product. The mean value of film thickness (n = 20) was the Z1 response, the uniformity of the coat (calculated as a standard deviation from film thickness) was the Z2 response, and the release of API in the buffer phase (in 130 min of dissolution test) was the Z3 response. A *p*-value of <0.05 was considered statistically significant. A full factorial plan for the fluid bed coating of MTs is described in Table 3.

## 3. Results and Discussion

### 3.1. Minitablet Cores

The direct compression of pantoprazole is problematic due to its sensitivity to the high temperatures that can be released during compression (tablets were observed to stick to the tools). However, wet granulation with alkaline salts protected pantoprazole and improved compressibility. In this way, pantoprazole was granulated with a 25% (*w*/*w*) solution of sodium carbonate [20]. In effect, both types of MT cores, MT-P with pantoprazole (25% *w*/*w*) and MT-D with diclofenac (15% *w*/*w*), were successfully manufactured in a rotary tablet press. Both types of the cores met the PhEur requirements for mass uniformity (MT-P 18 mg ± 10%; MT-D 21 mg ± 10%) and demonstrated good mechanical properties. The hardness values of 15.4 N (±1.24 N) for MT-P and 24.3 N (±3.56 N) for MT-D were measured. The friability test for all products produced values below 0.3%. Good mechanical strength, especially a low value in the friability test, is particularly important to ensure appropriate coating of MTs in a fluid bed.

### 3.2. Enteric-Coated Minitablets

Pantoprazole is known to be unstable in an acidic environment. Thus, contact had to be reduced between the active substance and methacrylic acid-ethyl acrylate copolymer (containing acid groups) during the coating process [20]. Separation of the enteric film from the cores was performed with a sub-coating (HPMC). Mass gains of the sub layer were observed at 12%. Diclofenac did not show similar interactions; therefore, the sub layer was not applied. For each experiment, a constant mass of enteric coating mixtures was used (30 g). Although both Eudragit L and Acryl Eze II contained 20% (*w*/*w*) dispersions of solids, they differed in their types of excipients and ratios. Therefore, slight differences in the setting process parameters were necessary since all parameters depended not only on the chosen polymer but also on the other ingredients in the coating mixtures. Thus, our purpose was to test different mixtures to better characterize the coating process and gastro-resistance of the obtained MTs.

Following the full factorial design, all parameter combinations were tested (Table 2). The yield of the coating process and thickness of the coat (factor Z1) were different for each experiment (Table 4) due to the use of various process parameters. Additionally, in some experiments, the coated MTs had a grainy surface. This aspect was described as a large standard deviation of film thickness in the response values. The standard deviation (SD) of the film thickness was marked as the Z2 factor. A loss of film uniformity may be a result of an undesired increase in the Z2 factor. However, not every process obtained a final product. In some experiments, the fluid was disturbed by nozzle blockages or inappropriate bed movements caused by MTs sticking to the tools (marked as a 0 value in Table 4). However, all results were taken into account in the statistical analysis.

### 3.3. In Vitro Dissolution Test

**Figure 2:**
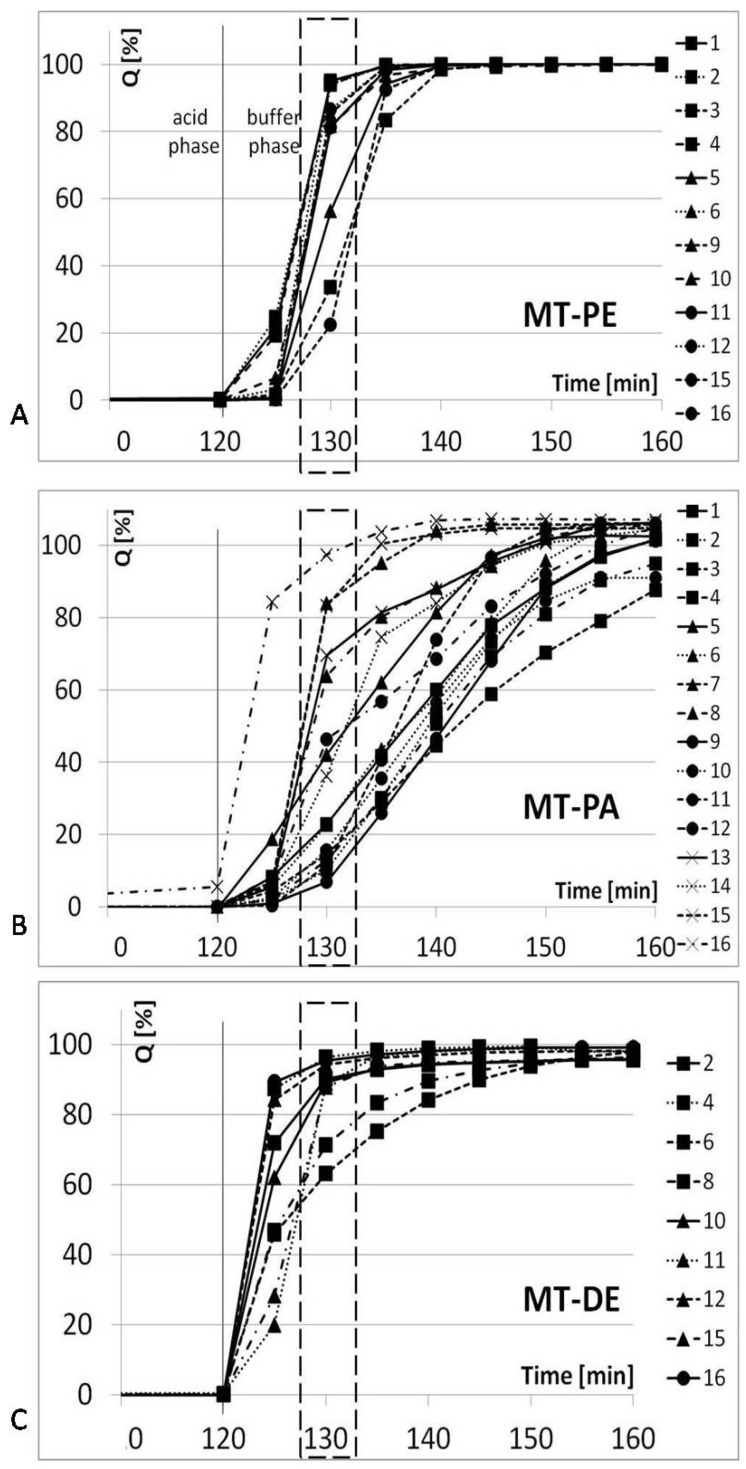
Dissolution profiles of enteric-coated MTs with pantoprazole ((**A**)—MT-PE; (**B**)—MT-PA) and diclofenac ((**C**)—MT-DE).

The PhEur dissolution test requirements were fulfilled by all tested MTs. The most significant variety of API release was observed at 130 min in the dissolution test for two products (23–95% for MT-PE and 7–85% for MT-PA). Therefore, the release of API at 130 min of dissolution test was chosen as the Z3 response value (Figure 2). The same time point was taken into consideration for MT-DE; although, in this case, a broad scattering of the results was observed at 125 min. Compared to MTs coated with Acryl Eze II, the units coated with Eudragit L showed a more reproducible release of API. This result may indicate increased sensitivity of Acryl Eze II under even slight changes to the parameters during the coating process. Therefore, when Acryl Eze II is used as a coating mixture, the process parameters should be strictly controlled to obtain reproducible dissolution test results and to produce MTs of the appropriate quality.

### 3.4. Statistical Analysis of the Coating Process

The design of a full factorial plan provides much more reliable and complete results than those obtained from screening experiments, so this type of design is especially useful in the early phases of experimental research. However, this process can be time-consuming, so the number of examined process factors should be ≤4. Therefore, to increase knowledge about the unexplored fluid bed coating processes of MTs, a two-level full factorial plan was chosen.

The quality of the final product and the enteric coating process yield were determined by all tested response values. The yield of the fluid bed coating process was evaluated by the thickness of the film (Z1), where the thicker the film, the higher the yield of the coating process. This relationship was produced by using the same amount of coating mixtures ineach experiment. The quality of the coated MTs was determined by two response values: Z2 (standard deviation of film thickness) and Z3 (the amount of API released after 120 min in acid and 10 min in the buffer stage). A low value of Z2 ensures uniformity of the coat and, consequently, gastro-resistance (no dissolution in acid stage), whereas a high value of Z3 produces a fast release of API in the buffer phase (pH 6.8). Therefore, high values of factors Z1 and Z3but low values of factor Z2 are desirable.

Experiments showed that some combinations of the parameters led to a final product with unsatisfactory quality or caused a failure of the coating process (marked as 0.0 in Table 2). Indicating the risky parameters that may cause process failure is a crucial part of optimizing the processes. Thus, it is vital to include in the statistical analysis all experiments that might have been responsible for the failure. However, this approach may affect the model fitting. Therefore, the coefficient of determination (R^2^) was counted to confirm model fitting. The R^2^ value ranged between 0 and 1 on the scale. Here, a higher value corresponds to higher predictive accuracy, stability, and quality of the models. Based on the literature [21], R^2^ > 0.67 indicatesa high predictive accuracy, a range of 0.33 < R^2^ < 0.67 indicates a moderate effect, while a range of 0.19 > R^2^ > 0.32 indicates a low effect. The R^2^ values calculated for these experiments showed mostly high and moderate effects (a low effect was obtained only for the MT-PA Z2 response). Therefore, an appropriate fit of the model was confirmed, and the results were considered reliable.

#### 3.4.1. Critical Process Parameters

**Figure 3:**
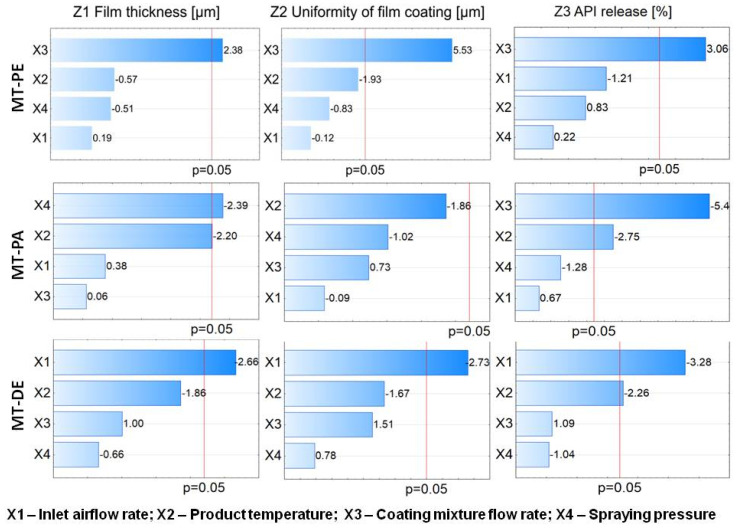
Pareto charts of the main effects for the fluid bed coating process of MTs.

The critical process parameters can be clearly identified using Pareto charts, where the effects influencing responses cross the red line [22]. The factors are listed in the graph according to the largest absolute standardized values. Figure 3 shows Pareto charts of the main effects of the tested parameters on responses.

Factor X3 (coating mixture flow rate) was significant for all response values tested for the MT-PE product. The impact of factor X3 on every response was positive, so by increasing the coating mixture flow rate, the response increased. The high value of Z1 and Z3 responses was related to an enhanced process yield and quality of the coated MTs, while a rise in the Z2 value was considered undesirable due to the possibility of forming an irregular coat with a grainy surface and a loss of gastro-resistance.

The analysis of the coating process performed for MT-PA showed that the tested independent variables influenced only Z1 (film thickness) and Z3 (API release) responses. In the Pareto charts, factor X2 (product temperature) was critical for both responses. This result reveals the sensitivity of the Acryl EZE II mixture to slight fluctuations in product temperature. The composition of the mixture, which determines the MFT (minimum film forming temperature), and product temperature may be other causes [23]. For the Z1 response, the X4 factor (spraying pressure) was also indicated as significant, and for the Z3 response, factor X3 was significant. All significant parameters had a negative impact: The higher the process parameter value used, the lower the value of every response achieved. This result means that Acryl EZE is more sensitive than the Eudragit L mixture to changing parameter settings during the process; for MT-PE only one factor was indicated as significant. 

However, research indicates the ease of film formation when coating with Acryl EZE II. A low value of parameter X4 causes the spraying of large drops of the coating mixture, which may result in the cores sticking together or forming an irregular film [24]. Instead, it was possible to obtain a thick and uniform film in a short time (none of the evaluated parameters affected the Z2 response). Additionally, slow spraying of the coating mixture facilitated a fast release of API. This result may be related to the thermal transformation of the polymer with other excipients of the coating mixture and the appropriate permeability of the film achieved during the coating process. For the MT-DE product, a significant X1 parameter (inlet airflow rate) was observed for all of the examined response values. Additionally, X2 (product temperature) was indicated as significant for the Z3 response. In all cases, the impact was negative. Compared to MT with pantoprazole (MT-P), the different masses, compositions, and geometries between the cores may be the reason for the different research results. Even small changes in the core characteristics can alter the dynamics of the fluid bed coating process [25]. In addition, the sub-coating used in MT-P can increase the core’s toughness and eliminate the effect of air flow rate on the coating process [26]. An intensive airflow rate can lead to abrasion of the cores and dusting. This is an undesirable phenomenon, as a large amount of dust in the chamber can lead to clogging of the nozzle, as well as the formation of a defective coat due to dust adsorption on the wetted cores. The use of a sub-coating improves the mechanical strength of the cores and protects against abrasion, thereby allowing the use of a higher inlet airflow rate at the stage of spraying the functional coating. Sub-coating also improves the adhesion of the film to the cores, which may facilitate the coating process and enhance the positive effects of the X3 factor (a high coating mixture flow rate leads to a thick but irregular film and a fast release of API). A similar relationship was observed for MT-DE without a sub-coating when a low value of X1 was used (a significant effect of the X1 instead of the X3 factor was noted).

#### 3.4.2. Optimization of Coating Process

**Figure 4:**
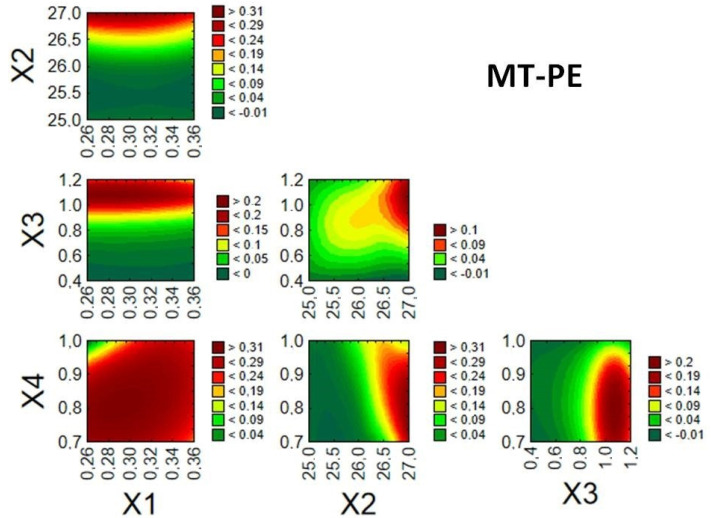
Desirability plots for response surface prediction of MT-PE showing interactions between factors X1–X4.

**Figure 5:**
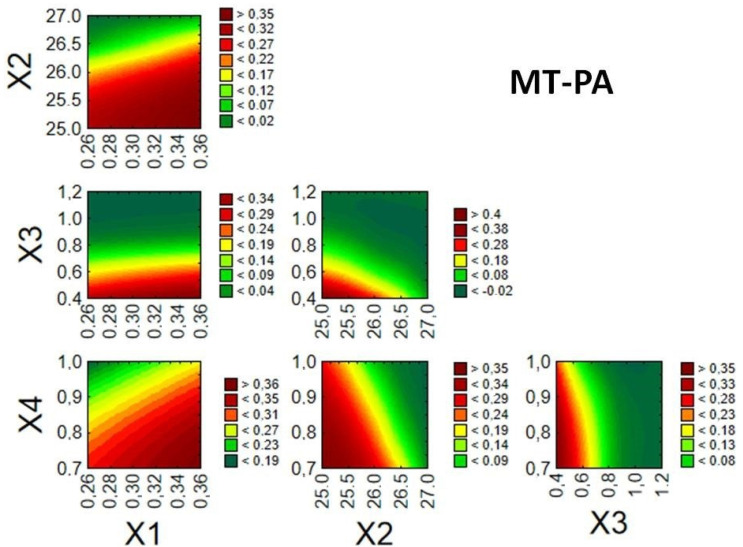
Desirability plots for response surface prediction of MT-PA showing interactions between factors X1–X4.

**Figure 6:**
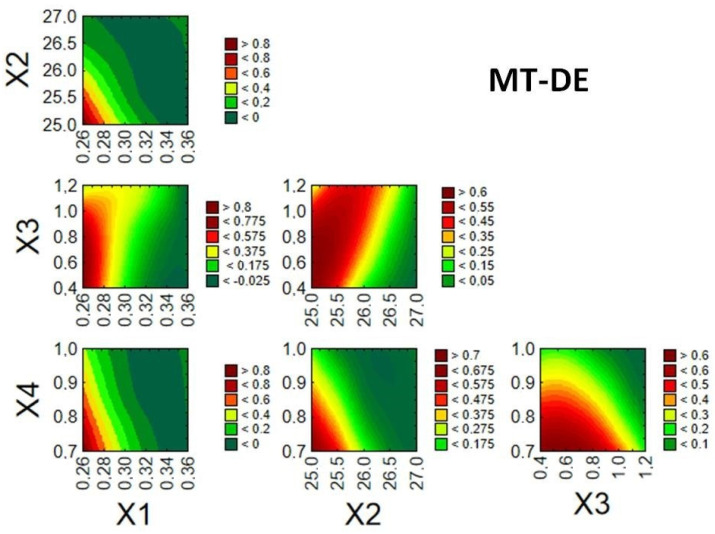
Desirability plots for response surface predictions of MT-DE showing the interactions between factors X1–X4.

Given the desired quality of the enteric MTs, it was possible to identify relationships between the examined factors and response values, thereby creating a design space and finding optimal conditions for the fluid bed coating process. Optimization of the processes was performed based on the desirability function proposed by Derringer and Suich [27]. Desired values of the dependent variables were designated as follows: Z1 (40–50 µm), Z2 (3–12 µm), and Z3 (60–100%). The splice–fit method was employed to create the design space for the fluid bed coating process of MTs (Figure 4, Figure 5 and Figure 6). Contour plots of desirability presented a design space. The large red space may be related to a stable process in the tested range. The colors were chosen automatically based on the established desirability (scale on the right). Desirability is related to the quality of the product. High desirability indicates a higher possibility to obtain the desired product. Further, the more values on the scale and colors in the graph, the more unstable the process.

In Figure 4, a large design space can be observed for factors X1 and X4. Thus, a flexible fluid bed coating process may be possible for these parameters over the whole tested range. However, the limited design space for X3 confirms that the process is sensitive to changes of this parameter.

A similar result was observed for MT-PA (Figure 5). Nearly the entire range of tested values for factors X1 and X4 enabled unrestricted execution of the coating process with high quality, thus creating a large design space. Although factor X4 was indicated as critical for the Z1 response, the prediction profile shows that this effect is more complex. Only the invalid setting of more than one parameter can result in disturbances.

Compared to previous products, it should be emphasized that a limited design space (red marks on graph) and stronger influence of the tested independent variables were observed for MT-DE (Figure 6). The broad colors and values on the scales shown in Figure 6 confirm that all coating processes of MTs were very responsive to changes of the tested parameters. However, all tested values of parameter X3 allowed the execution of a safe process and obtained a good-quality product.

**Figure 7:**
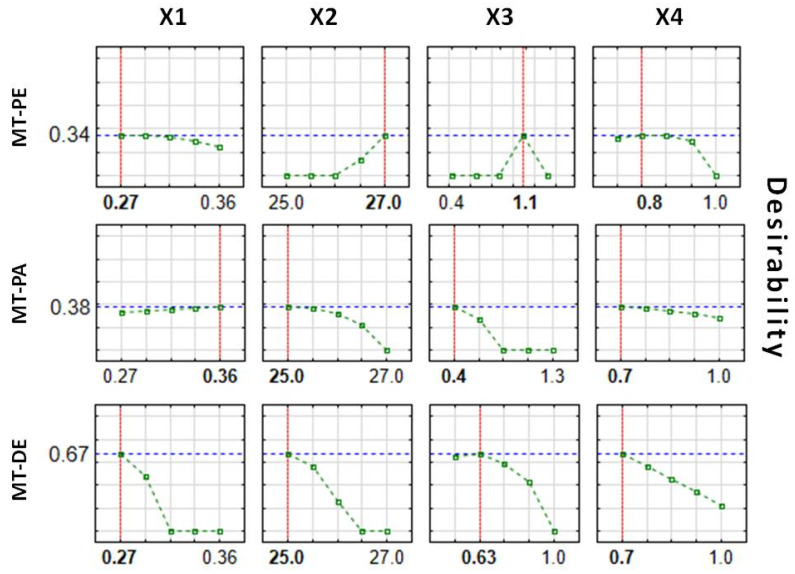
Predicted optimal values of factors X1–X4 and desirability of the fluid bed coating processes for all tested MTs.

Based on the defined ranges of the response values and their utility, the optimal factor values for the MT fluid bed coating process were determined (Figure 7). Moderate desirability can be obtained using MT-PE, and MT-PA quality values predicted using optimal parameters in the coating process (desirability > 0.3). However, the desirability value of >0.6 reached for MT-DE indicates the possibility to obtain high-quality coated MTs during the fluid bed coating process using optimal parameters. The results presented in Figure 7 indicate the inability to find process parameters that are optimal for all tested MTs. Although for MT-PA, a value of 0.36 m^3^/h is indicated here to be optimal (factor X1), the line of the effect is flat and close to the desirability value, which means that changes to this parameter in the tested range will not influence the product quality. The same was noted for the X4 factor for MT-PE, where 0.8 bar was indicated as optimal. However, the flat line was observed in the range of 0.7–0.8 bar. Therefore, for all products, the same predicted values of factors X1 and X4 were noticed to be optimal (X1 = 0.27 m^3^/h and X4 = 0.7 bar).

It can be concluded that lower values of parameters X1 and X4 could be successfully used in similar coating processes. However, the significant differences in parameters X2 and X3 emphasize the complexity of the coating process depending on conditions. When two commercial coating mixtures are compared (Acryl Eze II vs. Eudragit L), for MTs coated with Acryl Eze II (MT-PA), a decrease in the coating mixture flow rate (X3) is beneficial, which could be correlated with a decrease in the process temperature (X2). To obtain a good-quality product using Acryl Eze II, it is necessary to slow down the coating process and intensify control of the parameters. In the case of MT-DE, the lack of a sub-coating and different characteristics of the cores may also result in a longer coating process to obtain product of a high quality, which can be concluded from the lower optimal values of X2 and X3 factors in comparison to MT-PE. Therefore, optimization of the fluid bed coating process should be evaluated for individual conditions and product characteristics. However, checkpoint batches were not produced nor were stability data obtained, so optimization of the project has mostly predictive value.

## 4. Conclusions

The design of experiments as a tool for understanding the mechanisms of fluid bed coating allowed us to indicate the critical process parametersnecessary to determine the process yield and quality of the developed delayed release minitablets. Moreover, this process allowed us to find optimal values of the tested parameters. During the experiments, both high-quality and low-quality products were obtained, and in some cases no product was obtained. Different parameter settings allowed us to obtain a wide range of results. Therefore, this analysis may be valuable for technologists. The full factorial design indicated that changing certain details, such as the sub-coat, size, and mass of cores, as well as the core and coating mixture composition, may result in different parameters critical for the fluid bed coating process of MTs. The optimization of fluid bed coating allowed us to predict the values of process parameters useful to obtain a good-quality product. The results indicate that finding optimal parameters that are the same for all tested MTs is impossible. Therefore, DoE should be considered as a statistical tool to individually optimize the process parameter for the product and tested parameters. Even small changes, such as altering the composition of the coating mixture of the same polymer and changing the composition and mass of the cores with the same diameter, may result in different performance of the fluid bed coating process.

## Figures and Tables

**Table 1:** Characteristics of MT cores and process conditions.

	MTs with Pantoprazole (MT-P)	MTs with Diclofenac (MT-D)
API	25% pantoprazole sodium sesquihydrate	15% diclofenac sodium
Fillers	46.5% Vivapur PH102 17% Flowlac 100	40% Avicel PH101 25% GranuLac 200
Binder	3.5% sodium carbonate (dry weight)	10% Starch 1500
Disintegrant	4% Kollidon CLF	5% Vivastar P
Glidants	1% Aerosil 200 3% PRUV	2% Aerosil 200 3% PRUV
Unit mass	18 mg	21 mg
Impeller/Chopper speed	500/1000 rpm	600/1200 rpm
Rate of binder addition	5 mL/min	10 mL/min
Granulation time	20 min	15 min
Pressure force	100 MPa	200 MPa

**Table 2:** Characteristics of the fluid bed coating process: sub-coating and enteric coating (tested on two levels).

Factors	Process Parameters	Enteric Coating		Sub-Coating (Only MT-P)
		LOW	HIGH	
X1	Inlet airflow rate	0.27 m^3^/h	0.36 m^3^/h	0.36 m^3^/h
X2	Product temperature	25.0 °C	27.0 °C	43.0 °C
X3	Coating mixture flow rate	0.4 g/min	1.3 g/min	1.2 g/min
X4	Spraying pressure	0.7 bar	1.0 bar	1.1 bar
-	Coating time	75 min	23 min	50 min
-	Drying time	30 min		15 min
-	Amount of coating liquid	30 g		60 g

**Table 3:** A plan of the 2^4^ full factorial model.

No.	X1	X2	X3	X4
1	0.36	27.0	1.3	1.0
2	0.27	27.0	1.3	1.0
3	0.36	25.0	1.3	1.0
4	0.27	25.0	1.3	1.0
5	0.36	27.0	0.4	1.0
6	0.27	27.0	0.4	1.0
7	0.36	25.0	0.4	1.0
8	0.27	25.0	0.4	1.0
9	0.36	27.0	1.3	0.7
10	0.27	27.0	1.3	0.7
11	0.36	25.0	1.3	0.7
12	0.27	25.0	1.3	0.7
13	0.36	27.0	0.4	0.7
14	0.27	27.0	0.4	0.7
15	0.36	25.0	0.4	0.7
16	0.27	25.0	0.4	0.7

**Table 4:** The response values of full factorial design (16 experiments).

	Responses								
	MT-PE			MT-PA			MT-DE		
No.	Z1	Z2	Z3	Z1	Z2	Z3	Z1	Z2	Z3
1	44.8	9.6	95.1	35.7	10.0	22.9	0.0	0.0	0.0
2	42.4	10.9	93.9	42.0	8.9	10.3	48.1	12.4	90.1
3	59.3	22.2	33.7	40.0	11.9	14.6	0.0	0.0	0.0
4	52.0	13.8	94.1	49.4	10.9	12.9	57.5	25.5	96.5
5	51.8	6.2	56.4	32.6	7.1	42.2	0.0	0.0	0.0
6	49.4	2.9	94.4	38.9	8.6	22.7	43.4	7.0	63.1
7	0.0	0.0	0.0	52.6	12.3	84.0	0.0	0.0	0.0
8	0.0	0.0	0.0	41.2	11.1	63.9	37.4	8.0	71.3
9	51.3	6.3	85.4	49.3	11.3	7.0	0.0	0.0	0.0
10	57.3	19.1	81.8	41.8	11.2	15.7	47.0	7.4	89.2
11	64.6	22.2	81.4	50.7	11.1	11.5	55.3	8.8	88.7
12	61.5	22.1	86.6	47.2	10.2	46.4	44.7	6.7	94.3
13	0.0	0.0	0.0	49.9	10.1	69.7	0.0	0.0	0.0
14	0.0	0.0	0.0	44.0	11.3	36.3	0.0	0.0	0.0
15	60.1	5.9	22.5	48.3	9.4	83.3	51.1	6.7	87.9
16	51.8	5.7	81.3	47.4	11.6	84.5	40.8	6.7	95.4

Z1: Film thickness [µm]; Z2: Uniformity of film coating [µm]; Z3: API release [%].

## Data Availability

All data obtained during the study are available from the corresponding author on reasonable request.
